# Lymphoma-associated pancreatitis: a multicenter retrospective cohort study with a prediction model

**DOI:** 10.3389/fonc.2026.1853848

**Published:** 2026-09-30

**Authors:** Juju Feng, Qiwei Wang, Zhiyi Li, Tianjiao Duan, Aqian Zhao, Yanhui Zhang, Linqing Sun, Yun Feng

**Affiliations:** 1 Department of Spleen and Stomach Disease, Yulin Traditional Chinese Medicine Hospital, Yulin, Shaanxi, China; 2 Health Science Center, Yangtze University, Jingzhou, Hubei, China; 3 Department of Hematology, The First Affiliated Hospital of Xi’an Jiao Tong University, Xi’an, Shaanxi, China; 4 Department of Gastroenterology, Shaanxi Provincial People’s Hospital, Xi’an, Shaan Xi, China; 5 Department of Gastroenterology, The First Affiliated Hospital of Xi’an Jiao Tong University, Xi’an, Shaanxi, China

**Keywords:** EUS−FNA, L−asparaginase, lymphoma, pancreatitis, PET−CT, prediction model

## Abstract

**Purpose:**

Pancreatitis in patients with hematologic malignancies, particularly lymphoma, is a challenging complication with heterogeneous etiologies. Large studies with systematic etiological classification and prognostic models are lacking.

**Methods:**

This retrospective multicenter study (2020–2025) included patients with hematologic malignancies who developed acute pancreatitis. Etiologies were adjudicated using predefined criteria. A nomogram-based prediction model was developed with internal bootstrap validation.

**Results:**

Among 1,705 patients, 31 (1.82%) developed pancreatitis: 19 lymphoma (61.3%), 9 leukemia (29.0%), and 3 plasma cell disorders (9.7%). In the lymphoma cohort (median age 59 years, 57.9% male), diffuse large B-cell lymphoma (47.4%) and peripheral T-cell lymphoma (PTCL) (21.1%) were most common. Etiology distribution: treatment-related (36.8%), direct pancreatic infiltration (31.6%), conventional (15.8%), multifactorial (10.5%), and infection-related (5.3%). Endoscopic ultrasound (EUS)-guided tissue acquisition had 83.3% diagnostic sensitivity for direct infiltration; positron emission tomography/computed tomography (PET-CT) showed a “tetrad sign” in 5/6 cases. Severe pancreatitis occurred in 21.1% of lymphoma-associated pancreatitis. The nomogram (incorporating time to pancreatitis ≤30 days, recent L-asparaginase (LAsp)/immune checkpoint inhibitors (ICIs) exposure, and absence of pancreatic mass) discriminated treatment-related from non-treatment-related etiologies with AUC 0.84 (95% CI 0.71–0.96). PTCL subtype (HR 3.12, 95% CI 1.28–7.61, p=0.012), multifactorial etiology (HR 2.56, 95% CI 1.02–6.42, p=0.045), and age ≥65 years (HR 2.01, 95% CI 0.95–4.25, p=0.07) predicted 1-year mortality (C-index 0.79).

**Summary:**

Lymphoma-associated pancreatitis is rare (1.8% of hematologic malignancies). Treatment-related causes slightly outnumber direct infiltration. EUS-guided tissue acquisition and PET-CT are valuable for differential diagnosis. The nomogram may assist etiological classification and risk stratification, but prospective validation is required.

## 1 Introduction

Acute pancreatitis (AP) is a leading gastrointestinal reason for hospital admission, with gallstones and alcohol accounting for over 80% of cases (1). However, in patients with hematologic malignancies, pancreatitis may occur through several distinct mechanisms that can be categorized as: disease-related—neoplastic infiltration of the pancreas (primary or secondary lymphoma) or plasma cell disorders; treatment-related— chemotherapy-induced injury (particularly L-asparaginase (LAsp), but also cytarabine and vinca alkaloids) or targeted/immunotherapy-induced injury (immune checkpoint inhibitors, Bruton tyrosine kinase inhibitors, bispecific antibodies); infection-related— Epstein-Barr virus or cytomegalovirus; and conventional risk factors—gallstones, hypertriglyceridemia, hypercalcemia, and alcohol use (2–4).

Primary pancreatic lymphoma accounts for <2% of extranodal lymphomas and ~0.2% of pancreatic tumors (5, 6). Diffuse large B-cell lymphoma is the most common subtype. Endoscopic ultrasound-guided fine-needle aspiration (EUS-FNA) diagnostic accuracy may be inadequate, and diagnosis often requires surgical resection. Endoscopic ultrasound-guided fine-needle biopsy (EUS-FNB) improves accuracy by providing tissue for immunohistochemistry (7, 8). Fluorodeoxyglucose-positron emission tomography/computed tomography is also useful, though primary pancreatic lymphoma can mimic carcinoma (9). Secondary pancreatic involvement rarely presents as AP.

L-asparaginase-associated pancreatitis (AAP) is well described in pediatric acute lymphoblastic leukemia (ALL). A multicenter study of 7,640 children reported 3.9% incidence; age ≥10 years and intermediate/high-risk status were risk factors (10). Data in adult lymphoma patients are scarce.

Immune checkpoint inhibitors (ICIs) increase pancreatitis risk (11–13). Preemptive treatment allows rechallenge in some patients. Bruton tyrosine kinase inhibitors also have been associated with pancreatic adverse events, though the risk appears to vary by agent (13). First-generation covalent Bruton tyrosine kinase inhibitors (ibrutinib) have been most frequently associated with pancreatitis (12). Second-generation covalent inhibitors (acalabrutinib, zanubrutinib) have improved selectivity for Bruton tyrosine kinase and may have a lower risk of off-target pancreatic toxicity (13), though case reports of pancreatitis have been documented. Data on non-covalent Bruton tyrosine kinase inhibitors (e.g., pirtobrutinib) are currently limited (13).

Plasma cell disorders rarely involve the pancreas as extramedullary plasmacytoma, which carries a poor prognosis with median survival <15 months (14, 15).

Given the lack of large multicenter studies, we aimed to (1) describe the frequency, clinical characteristics, and etiological spectrum of pancreatitis in lymphoma vs. leukemia and plasma cell disorders; (2) evaluate endoscopic ultrasound-guided fine needle aspiration/biopsy (EUS-FNA/FNB) and positron emission tomography/computed tomography (PET-CT) for differential diagnosis; (3) evaluate management and rechallenge; and (4) develop a nomogram for etiology and prognosis in lymphoma-associated pancreatitis (LAP).

## 2 Materials and methods

### 2.1 Study design and setting

This retrospective cohort study included four tertiary centers from January 2020 to December 2025. The study was approved by Ethics Committee of The First Affiliated Hospital of Xi’an Jiao Tong University (XJTUAE2025-2108) and complied with the Declaration of Helsinki.

### 2.2 Participants

We included patients with pathologically confirmed hematologic malignancy who developed AP according to the revised Atlanta criteria (16) (abdominal pain, amylase/lipase ≥3×upper limit of normal, or imaging findings). Chronic pancreatitis without acute exacerbation was excluded. Management followed the International Association of Pancreatology revised guidelines (17) and Chinese 2021 guidelines (18). Lymphoma histologies were classified according to the WHO 2016/2022 classification (19) and included diffuse large B-cell lymphoma, PTCL not otherwise specified, anaplastic lymphoma kinase-negative anaplastic large cell lymphoma, angioimmunoblastic T-cell lymphoma, follicular lymphoma, natural killer/T-cell lymphoma, and others (mucosa-associated lymphoid tissue lymphoma, mantle cell lymphoma). Leukemia types included ALL and acute myeloid leukemia. Plasma cell disorders included multiple myeloma and solitary plasmacytoma.

### 2.3 Study flow diagram

**Figure 1** summarizes patient selection. Of 1,705 hematologic malignancy patients, 31 (1.82%) had AP: 19 lymphoma, 9 leukemia, 3 plasma cell disorders. Among the 19 lymphoma patients, etiological classification yielded direct infiltration (n=6), treatment-related (n=7), conventional (n=3), infection-related (n=1), and multifactorial/idiopathic (n=2).

**Figure 1:**
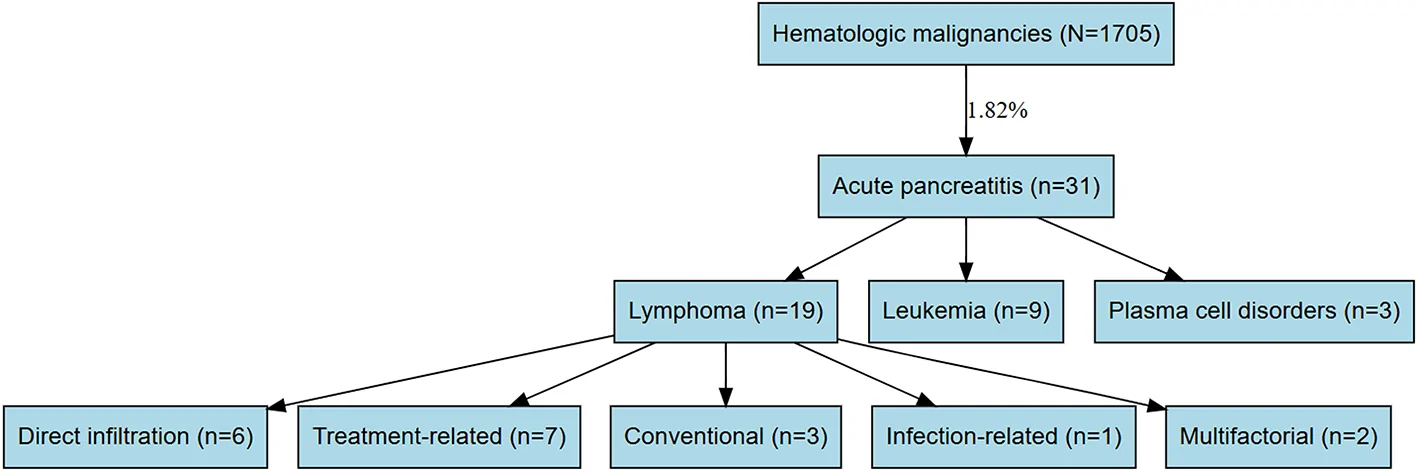
Study flow diagram. HL, hematologic malignancies; AP, acute pancreatitis; EUS-FNA/FNB, endoscopic ultrasound-guided fine needle aspiration/biopsy; PET-CT, positron emission tomography/computed tomography; LAP, lymphoma-associated pancreatitis.

### 2.4 Data collection

We extracted comprehensive clinical data including demographics, lymphoma subtype, stage, International Prognostic Index, and treatments details (regimens, timing, and specific medications). Laboratory data included pancreatic enzymes, lipids, calcium, and viral serologies. Imaging findings (computed tomography, magnetic resonance imaging, EUS, and PET-CT), pathology results, pancreatitis severity, complications, management strategies, therapy modifications, and follow-up outcomes were also recorded.

### 2.5 Diagnostic evaluation of pancreatic involvement

EUS-FNA/EUS-FNB. Performed with 22G or 19G needles. Diagnostic yield defined as sufficient tissue for definitive diagnosis.

PET-CT. Qualitative and semi-quantitative (SUVmax) analysis. Typical imaging features of pancreatic lymphoma defined were as a study-derived composite “tetrad sign”, based on previously reported imaging characteristics (7, 9): homogeneous fluorodeoxyglucose (FDG) uptake; absent or mild ductal dilation disproportionate to tumor size; preserved peri-pancreatic fat planes; and associated lymphadenopathy with similar FDG avidity. These features are suggestive but not pathognomonic for pancreatic lymphoma, as FDG uptake patterns can be observed in many pancreatic entities (20). PET-CT images were reviewed independently by two experienced radiologists (one nuclear medicine specialist and one diagnostic radiologist), both blinded to the clinical data. In cases of discrepancy (defined as disagreement on the presence or absence of any of the four tetrad sign features), a consensus reading was performed with a third senior radiologist, and the majority opinion was recorded.

### 2.6 Etiological adjudication

A panel of two hematologists and one gastroenterologist assigned primary etiology (detailed criteria in **Supplementary Table S1**):

Direct tumor infiltration: biopsy-proven or imaging lesion with regression on lymphoma therapy.

Treatment-related: onset ≤ 14 days of LAsp, cytarabine, or vinca alkaloids; or ≤ 30 days of bruton tyrosine kinase inhibitor, ICI, or bispecific antibody (in our cohort, the bispecific antibody was blinatumomab in one case). The 30-day window for ICI-related pancreatitis was chosen based on published literature reporting that ICI-related pancreatic injury typically occurs within the first 2–16 weeks of treatment initiation, with a median time to onset of approximately 57 days (11, 13). This conservative threshold was selected to maximize sensitivity for detecting potential ICI-related cases.

Infection-related: active Epstein-Barr virus/cytomegalovirus viremia with temporal association.

Conventional: gallstones, triglycerides > 11.3 mmol/L, corrected calcium > 2.75 mmol/L, or heavy alcohol use(defined as > 50 g/day for men and > 40 g/day for women).

Multifactorial/idiopathic: ≥ 2 plausible causes or none identified.

### 2.7 Development of prediction model

The prediction model had two primary outcomes: treatment-related versus non-treatment-related etiology, assessed using multivariable logistic regression, and 1-year all-cause mortality, assessed using Cox proportional hazards regression.

Candidate predictors were selected based on literature review and clinical plausibility, including age, sex, lymphoma subtype, time from lymphoma diagnosis to pancreatitis (≤ 30 days), lactate dehydrogenase ratio, B symptoms, International Prognostic Index, prior lines of therapy, pancreatitis severity, intensive care unit admission, necrosis, and etiology category.

Model development was performed using logistic regression with stepwise selection based on the Akaike information criterion, and Cox regression with backward selection (entry *p* < 0.10, removal *p* > 0.05). Nomograms were constructed using the rms package in R version 4.2. Internal validation was conducted with bootstrap resampling (500 replicates). Model performance was assessed using the area under the curve (AUC) for the logistic model, Harrell’s C-index for Cox model, and calibration evaluated using the Hosmer-Lemeshow test and calibration plots.

### 2.8 Statistical analysis

Continuous variables were expressed as median (interquartile range)and compared using the Mann-Whitney U test or Kruskal-Wallis test, as appropriate. Categorical variables were compared using the chi-square or Fisher’s exact test. Overall Survival was estimated using the Kaplan-Meier method, and group comparisons were performed using the log-rank test. All tests were two-sided, and a *p*-value < 0.05 was considered statistically significant. Statistical analyses were performed using SPSS 26.0 and R version 4.2.

## 3 Results

### 3.1 Patient characteristics

Of 1,705 hematologic malignancy patients, 31 (1.82%) had AP: lymphoma 19 (61.3%), leukemia 9 (29.0%), plasma cell 3 (9.7%). The lymphoma cohort (**Table 1**) had median age 59 years (interquartile range 45-71), 57.9% male. Diffuse large B-cell lymphoma (47.4%) and peripheral T-cell lymphoma (PTCL) (21.1%) were most common. Advanced stage (III-IV) was 73.7%.

**Table 1:** Baseline characteristics of lymphoma patients with pancreatitis (n = 19).

Characteristic	Value
Age, median (interquartile range), years	59 (45–71)
Male sex, n (%)	11 (57.9)
Lymphoma subtype	
Diffuse large B-cell lymphoma, n (%)	9 (47.4)
PTCL, n (%)	4 (21.1)
Follicular, n (%)	2 (10.5)
NK/T-cell, n (%)	2 (10.5)
Others, n (%)	2 (10.5)
Stage III-IV, n (%)	14 (73.7)
International Prognostic Index score ≥3, n (%)	9 (47.4)
Prior chemotherapy lines, median (interquartile range)	1 (0–2)
Time from lymphoma diagnosis to pancreatitis, months, median (interquartile range)	3.5 (0.5–12.8)

PTCL, peripheral T-cell lymphoma.

### 3.2 Etiological classification of LAP

**Figure 2** shows etiology: treatment-related (7/19, 36.8%), direct infiltration (6/19, 31.6%), conventional (3/19, 15.8%), multifactorial (2/19, 10.5%), infection-related (1/19, 5.3%). Among treatment-related, LAsp accounted for 4 (57.1%), PD-1 inhibitors 2, ibrutinib 1. Conventional causes: biliary stones (2), hypertriglyceridemia (1).

**Figure 2:**
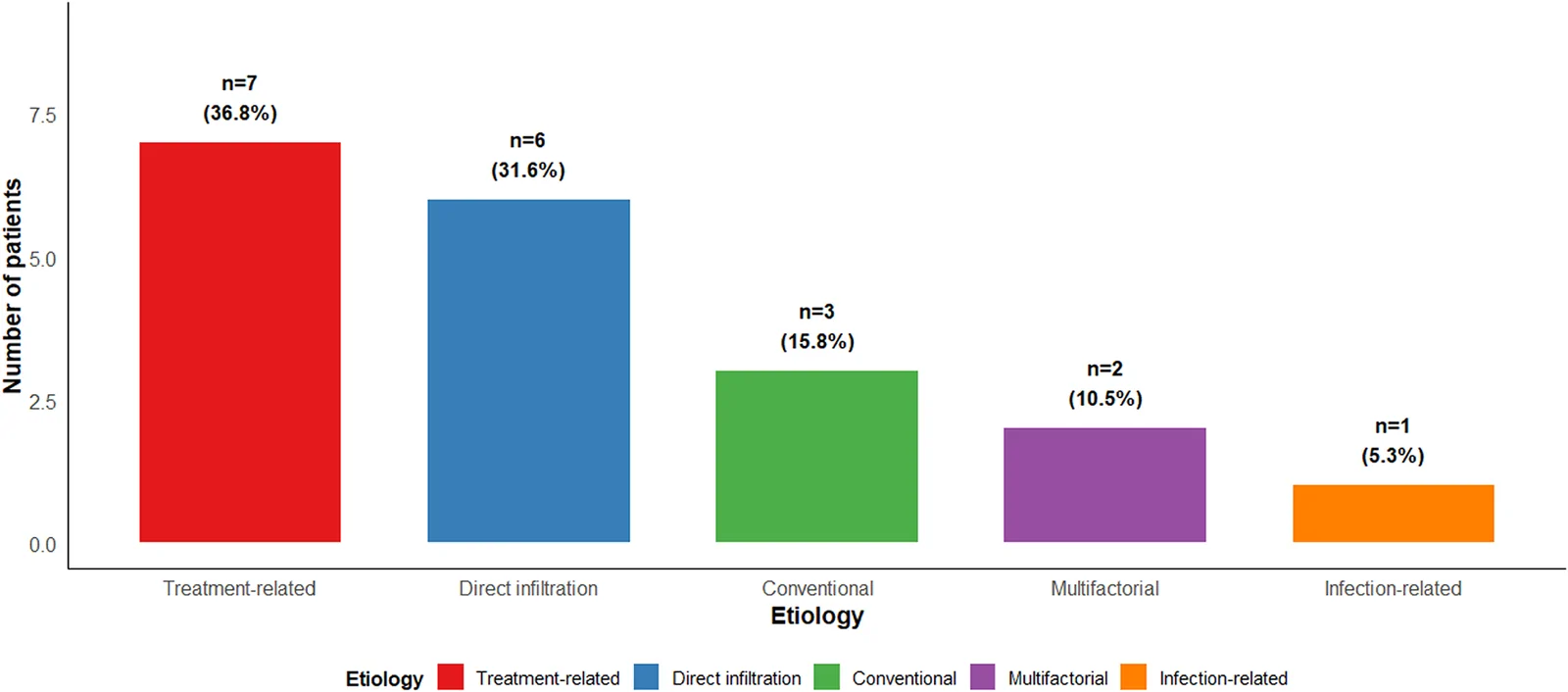
Etiological distribution of LAP (n = 19). Bar chart showing the distribution of etiological categories among 19 patients with lymphoma-associated pancreatitis. LAP, lymphoma-associated pancreatitis.

### 3.3 Diagnostic utility of EUS-FNA/FNB and PET-CT

Among the 6 patients with direct infiltration, the median age was 62 years (range 51–74), and 4 were male. All 6 patients presented with abdominal pain, and 3 had associated weight loss. Imaging revealed a pancreatic mass in all cases (median size 4.2 cm, range 2.5–7.0 cm), with the head of the pancreas involved in 4 cases and the body/tail in 2 cases. EUS-FNA or EUS-FNB was performed in all 6 cases, yielding sufficient tissue for histopathological diagnosis in 5 cases (sensitivity 83.3%). Immunohistochemistry confirmed diffuse large B-cell lymphoma in 4 cases, follicular lymphoma in 1 case, and peripheral T-cell lymphoma in 1 case. All 6 patients received lymphoma-directed therapy (rituximab + cyclophosphamide + doxorubicin + vincristine + prednisone for diffuse large B-cell lymphoma, n=3; cyclophosphamide + doxorubicin + vincristine + etoposide + prednisone for peripheral T-cell lymphoma, n=2; rituximab + bendamustine for follicular lymphoma, n=1), with 5 patients (83.3%) achieving resolution of the pancreatitis and 4 patients (66.7%) achieving complete or partial remission of the lymphoma at 6-month follow-up.

PET-CT was performed in 5 patients and showed the tetrad sign (homogeneous uptake 5/5, no ductal dilation 4/5, preserved fat planes 5/5, associated lymphadenopathy 4/5). The median SUVmax was 8.4. In contrast, among patients with treatment-related pancreatitis (n=7), no focal pancreatic masses or abnormal FDG uptake was observed on PET-CT.

### 3.4 Clinical presentation and severity

Abdominal pain was the most common presenting symptom, occurring in 89.5% of patients, followed by nausea/vomiting (68.4%) and fever (42.1%). The median peak serum lipase was 1,156 U/L (interquartile range 498-2,876). According to the revised Atlanta classification, the majority of patients had mild (42.1%) or moderate (36.8%) pancreatitis, while 21.1% developed severe pancreatitis. Pancreatic necrosis was observed in 10.5% of cases, pseudocyst formation in 5.3%, and persistent organ failure in 15.8%. Intensive care unit admission was required in 26.3%of patients.

### 3.5 Management and therapy modifications

Supportive care followed guidelines. Endoscopic retrograde cholangiopancreatography in 2 biliary cases. Offending drug permanently discontinued in 5/7 treatment-related patients. Among 4 with mild-to-moderate AAP, 2 (50%) were rechallenged without recurrence. Ibrutinib switched to acalabrutinib without recurrence. Direct infiltration patients received lymphoma therapy without delay in 5/6.

### 3.6 Outcomes in lymphoma cohort

Recovery without sequelae in 78.9%. Recurrent pancreatitis in 10.5% (median follow-up 20 months). 90-day mortality 10.5%, 1-year all-cause mortality 15.8%. PTCL subtype (HR 3.12, *p* = 0.012) was the strongest predictor.

### 3.7 Subgroup analyses

Subgroup analyses were performed for patients with leukemia and plasma cell disorders. Among the 9 leukemia patients, 7 had ALL and 2 had acute myeloid leukemia. The incidence of AAP in ALL patients was 5.9% (7/119). Age ≥10 years was identified as a significant risk (OR 3.5, *p* = 0.04). Among 3 patients with mild AAP who underwent rechallenge with LAsp, 2 (66.7%) tolerated the re-exposure without recurrence. In the plasma cell disorder group, 2 patients presented with pancreatic plasmacytoma, with a median survival of 9 months, while 1 patient developed hypercalcemia-induced pancreatitis without structural pancreatic lesions.

### 3.8 Comparison across groups

**Table 2** compares lymphoma, leukemia, and plasma cell groups. Lymphoma patients were older (*p* < 0.001) and had more direct infiltration (31.6% vs 0%, *p* = 0.04). Leukemia had predominantly treatment-related (77.8%). 90-day mortality was 10.5%, 11.1%, and 33.3%, respectively (*p* = 0.46).

**Table 2:** Comparison of pancreatitis across hematologic malignancy groups.

Feature	Lymphoma (n = 19)	Leukemia (n = 9)	Plasma cell (n = 3)	*p* value
Age, median (interquartile range), years	59 (45–71)	10 (6–13)	68 (62–74)	<0.001
Male sex, n (%)	11 (57.9)	5 (55.6)	2 (66.7)	0.94
Severe pancreatitis, n (%)	4 (21.1)	1 (11.1)	1 (33.3)	0.68
Intensive care unit admission, n (%)	5 (26.3)	2 (22.2)	1 (33.3)	0.91
Pancreatic necrosis, n (%)	2 (10.5)	1 (11.1)	0 (0)	0.84
Primary etiology, n (%)		—		0.04
Direct infiltration	6 (31.6)	0 (0)	2 (66.7)	
Treatment-related	7 (36.8)	7 (77.8)	0 (0)	
Conventional	3 (15.8)	1 (11.1)	1 (33.3)	
Other	3 (15.8)	1 (11.1)	0 (0)	
90-day mortality, n (%)	2 (10.5)	1 (11.1)	1 (33.3)	0.46

### 3.9 Prediction model

#### 3.9.1 Treatment-related vs. non-treatment-related etiology

To further explore the determinants of treatment-related pancreatitis, we performed multivariable logistic regression analysis. Multivariable logistic regression identified time to pancreatitis ≤30 days (OR 6.8, 95% CI 1.2-38.5, *p* = 0.03), recent LAsp/ICI exposure (OR 12.4, 95% CI 1.8-85.3, *p* = 0.01), and absence of pancreatic mass (OR 5.2, 95% CI 0.9-30.1, *p* = 0.06) as independent predictors (**Table 3**). The nomogram (**Figure 3A**) had bootstrap-corrected AUC 0.84 (95% CI 0.71-0.96), with the ROC curve shown in **Supplementary Figure S1**. Calibration was satisfactory (Hosmer-Lemeshow test *p* = 0.38, **Supplementary Figure S2**). The Brier score for the model was 0.18, indicating good overall performance.

**Table 3:** Multivariable predictors in the prediction models.

Model/Predictor	β coefficient	OR/aHR	95% CI	*p* value
Treatment-related etiology (logistic)				
Time to pancreatitis ≤30 days	1.92	6.8	1.2-38.5	0.03
Recent LAsp/ICI exposure	2.52	12.4	1.8-85.3	0.01
Absence of pancreatic mass	1.65	5.2	0.9-30.1	0.06
1-year mortality (Cox)				
PTCL subtype	1.14	3.12	1.28-7.61	0.012
Multifactorial etiology	0.94	2.56	1.02-6.42	0.045
Age ≥65 years	0.70	2.01	0.95-4.25	0.07

LAsp, L-asparaginase; ICI, immune checkpoint inhibitor; PTCL, peripheral T-cell lymphoma.

**Figure 3:**
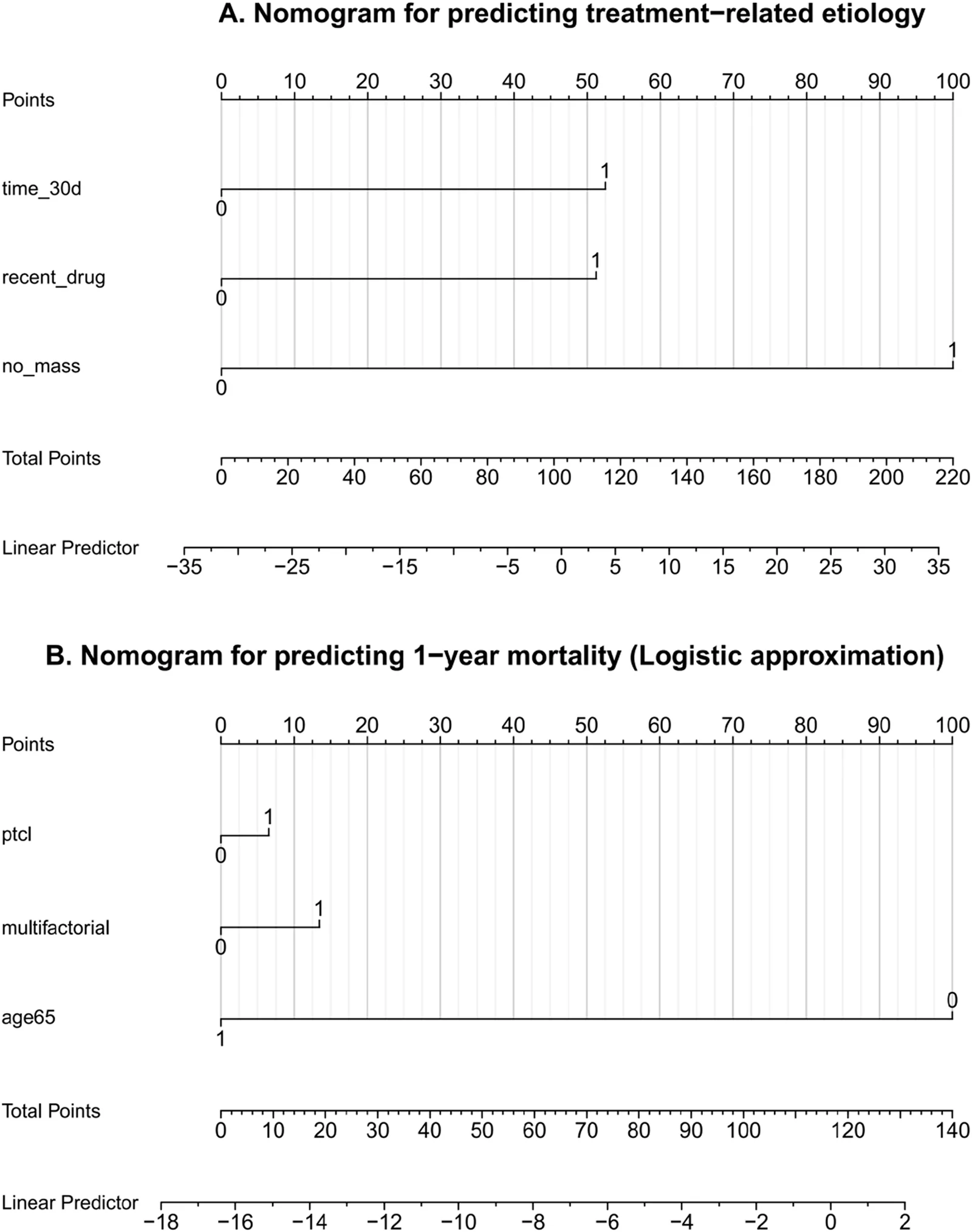
Nomogram for predicting treatment-related etiology and 1-year mortality. **(A)** Nomogram for predicting treatment-related vs. non-treatment-related etiology. Points: time to pancreatitis ≤30 days (20 points), recent L-asparaginase (LAsp)/immune checkpoint inhibitors (ICI) exposure (30 points), absence of pancreatic mass (10 points). Total points to predicted probability. **(B)** Nomogram for predicting 1-year mortality. Points: peripheral T-cell lymphoma (PTCL) subtype (30 points), multifactorial etiology (20 points), age ≥65 years (10 points). Total points: ≥4 = high risk (>30% mortality), 2-3 = intermediate risk (10-30%), ≤1 = low risk (<10%).

#### 3.9.2 1-year mortality

For the prediction of 1-year mortality, we performed Cox proportional hazards regression analysis. PTCL subtype (HR 3.12, *p* = 0.012), multifactorial etiology (HR 2.56, *p* = 0.045), and age ≥65 years (HR 2.01, *p* = 0.07) were independent predictors (**Table 3**). The prognostic nomogram (**Figure 3B**) had C-index 0.79 (95% CI 0.67-0.91).

## 4 Discussion

This multicenter retrospective study provides, to our knowledge, a systematic analysis of pancreatitis in patients with hematologic malignancies with comparative data across lymphoma, leukemia, and plasma cell disorders, incorporating evaluation of the diagnostic utility of EUS-FNA/FNB and PET-CT, and a nomogram-based prediction model for etiology and prognosis in LAP. The study addresses a significant clinical gap, as management decisions differ markedly between treatment-related pancreatitis (drug discontinuation, supportive care) and direct tumor infiltration (prompt anti-lymphoma therapy).

Pancreatitis in patients with hematologic malignancies is uncommon but not negligible, with an overall incidence of 1.82% (31/1,705) in our cohort. This is consistent with previous estimates in the literature (21, 22). Among the three malignancy groups, lymphoma accounted for the majority of cases (61.3%), reflecting both the higher prevalence of lymphoma in adult populations and the diversity of etiological mechanisms.

The etiological spectrum differs markedly across malignancy types. In lymphoma patients, treatment-related causes (36.8%) slightly outnumbered direct tumor infiltration (31.6%). This finding challenges the assumption that most cases are due to drug toxicity alone. In routine practice, clinicians might default to drug-related attribution without adequately excluding pancreatic lymphoma or conventional causes. Our systematic adjudication revealed that conventional causes (biliary stones, hypertriglyceridemia) accounted for 15.8% of cases, emphasizing the need for thorough diagnostic workup, including abdominal ultrasound and lipid profile, before attributing pancreatitis to lymphoma or its treatment. In contrast, leukemia patients overwhelmingly presented with treatment-related pancreatitis (77.8%), almost exclusively LAsp-associated, reflecting the central role of this agent in ALL therapy. The incidence of AAP in ALL patients in our study (5.9%) aligns with the published range of 2–10%, with the largest multicenter study of 7,640 Children ALL patients reporting an AAP incidence of 3.9% (10). The slightly higher incidence in our cohort may reflect differences in patient population and treatment protocols. Previous studies have shown that pancreatitis is more prevalent among elderly patients who have received treatment with asparaginase (22, 23). Plasma cell disorder patients uniformly presented with either pancreatic plasmacytoma or hypercalcemia-induced pancreatitis, representing distinct and rare mechanisms (14, 20).

The diagnostic evaluation of pancreatic involvement in lymphoma patients remains challenging. Our study demonstrates that EUS-FNA/EUS-FNB achieved a diagnostic sensitivity of 83.3% for direct pancreatic involvement. The literature reports an overall accuracy of EUS-FNA for pancreatic masses of approximately 85–90% (24). Importantly, EUS-FNB has emerged as the preferred technique for sampling solid pancreatic lesions, as it delivers larger tissue cores and preserves histological architecture, thereby enhancing diagnostic accuracy and facilitating personalized treatment (25, 26). A recent meta-analysis comparing auxiliary techniques (contrast-enhanced EUS or elastography) to standard EUS-FNA/B found that while EUS-FNA/B has high specificity, sensitivity remains less than 90% without rapid on-site evaluation, and auxiliary techniques may improve diagnostic outcomes (27, 28). In pancreatic lymphoma specifically, where immunohistochemical analysis is critical for subtyping, the adoption of EUS-FNB with larger-caliber needles and tissue acquisition for paraffin embedding is particularly advantageous. In our series, the single non-diagnostic case was due to sampling of a predominantly necrotic area, highlighting the importance of targeting viable tumor tissue. No procedure-related complications were observed, supporting the safety of EUS-guided sampling in this patient population.

PET-CT also proved valuable in our cohort. In our study, the four imaging features—homogeneous FDG uptake, absence of pancreatic ductal dilation disproportionate to tumor size, preservation of peri-pancreatic fat planes, and associated lymphadenopathy with similar FDG avidity— were observed in 80–100% of cases. These features, which we term the “tetrad sign” as a study-defined composite, are consistent with previous reports (7, 9). However, it is crucial to recognize that FDG uptake patterns in are nonspecific (20); therefore, PET-CT findings alone are insufficient for definitive diagnosis and should always be complemented by pathological confirmation. The absence of focal pancreatic lesions or abnormal FDG uptake on PET-CT in patients with treatment-related pancreatitis helped exclude tumor infiltration, supporting the role of PET-CT as a non-invasive screening tool to differentiate between these etiologies when EUS-FNA is not feasible or yields non-diagnostic results.

LAsp rechallenge after AAP requires careful individualization. In our cohort, successful rechallenge was observed in some lymphoma and leukemia patients with mild AAP, consistent with previous reportsliter (10, 22). While pediatric among rechallenge may improve outcomes in selected patients, particularly those with intermediate/high-risk ALL, studies have reported secondary AAP recurrence rates of 30-50% (10, 29, 30), suggesting that rechallenge decisions must be made on a case-by-case basis. Recent studies have developed predictive models for AAP severity in pediatric ALL, identifying age, C-reactive protein, and fibrinogen as independent predictors (31, 32). These models focus on severity prediction within a specific drug-induced pancreatitis cohort, whereas our model addresses etiology classification in lymphoma patients, highlighting complementary clinical applications.

For ICI-associated pancreatitis, our observation of 2 cases (10.5% of LAP) is consistent with published reports (11). Meta-analyses have confirmed that ICI therapy increases the risk of any-grade pancreatitis (33), and rechallenge appears feasible in selected patients after recovery (13, 34, 35). However, some patients may develop delayed exocrine or endocrine insufficiency, underscoring the need for follow-up (13). These findings support monitoring pancreatic enzymes during ICI therapy and considering rechallenge after appropriate preemptive management in patients with prior ICI-related pancreatitis.

Pancreatic involvement in plasma cell disorders carries a grim prognosis. The two patients with pancreatic plasmacytoma in our series had a median survival of 9 months. Extramedullary plasmacytoma involving the pancreas is uncommon and often mimics pancreatic cancer, and is associated with a poor prognosis (36). These cases underscore the importance of considering pancreatic plasmacytoma when a patient with known multiple myeloma presents with a pancreatic mass, particularly one showing rapid interval growth, and the need for multidisciplinary team management.

The prognosis of LAP is driven more by lymphoma biology than by pancreatitis severity per se. PTCL subtype independently predicted 1-year mortality (HR 3.12), reflecting the aggressive nature of T-cell lymphomas. The 5-year survival for PTCL patients remains suboptimal, and the occurrence of pancreatitis may further complicate treatment delivery. The addition of a prediction model to clinical practice could assist in early identification of high-risk patients who may benefit from more aggressive supportive care or modified treatment protocols.

The nomogram-based prediction model developed in this study achieved good discriminative ability for both etiology classification (AUC 0.84) and 1-year mortality prediction (C-index 0.79). The three key predictors for etiology—time to pancreatitis ≤30 days, recent exposure to LAsp/ICI, and absence of pancreatic mass—are readily available in clinical practice and can guide decision-making. For example, a patient presenting with pancreatitis within 30 days of lymphoma diagnosis without an identifiable pancreatic mass on imaging and with recent LAsp exposure has a high probability of treatment-related etiology, allowing clinicians to focus on supportive care and consider drug rechallenge rather than pursuing invasive diagnostic procedures. The prognostic nomogram identifies PTCL subtype as the strongest predictor, followed by multifactorial etiology and advanced age. This tool can assist in risk stratification for clinical trials and guide intensity of supportive care. From a clinical utility perspective, the model can be easily applied at the bedside using the simplified scoring system, facilitating rapid triage of patients into low-, intermediate-, or high-risk categories for treatment-related pancreatitis and mortality. Future directions for model improvement include external validation in larger independent cohorts, decision curve analysis to quantify clinical net benefit at various threshold probabilities, and potentially the development of a web-based risk calculator to facilitate bedside clinical decision-making.

This study has several limitations. First, the retrospective design carries inherent selection and information bias. Second, despite multicenter collaboration, the absolute number of cases (especially in the plasma cell disorder group) is small, limiting statistical power for subgroup analyses and model validation. Third, no central pathology or imaging review was performed, and not all cases of suspected direct pancreatic infiltration were confirmed by biopsy. In some cases, the diagnosis was based on imaging features and response to lymphoma therapy rather than pathological confirmation. Fourth, we did not systematically collect data on hepatitis virus (Hepatitis B virus, Hepatitis C virus) serologies, which may be relevant in the context of hematologic malignancies and pancreatitis. This represents a limitation of our retrospective data collection. Fifth, we did not collect genetic risk variants (e.g., UGT1A1, CPA2) that may predispose to AAP. Sixth, the prediction model was developed on a very small sample (19 lymphoma-associated pancreatitis cases, with only 7 treatment-related and 6 direct infiltration cases). The number of events per variable in the logistic and Cox regression models is substantially below the recommended threshold of 10–20 events per predictor variable (37, 38). Consequently, the model is at high risk of overfitting, and the reported AUC (0.84) and C-index (0.79) represent optimism-corrected internal estimates that may not generalize to external populations. Therefore, our nomogram should be considered exploratory and hypothesis-generating rather than a clinically validated tool. External validation in larger independent cohorts is mandatory before any clinical application. – center Seventh, the generalizability to low-income settings where LAsp monitoring is less accessible may be limited.

Future directions include prospective registries with standardized etiological workup. Biomarkers to distinguish pancreatic infiltration from drug-induced injury—such as circulating tumor DNA for lymphoma-associated mutations (e.g., MYD88 L265P, EZH2), cytokine profiling (IL-6, IL-8, C-reactive protein dynamics), and proteomics combined with machine learning—could aid real-time decisions. For LAsp, pharmacokinetic-guided dosing and concomitant protease inhibitors warrant investigation to reduce AAP risk. The role of early PET-CT in detecting subclinical pancreatic involvement should be explored in larger prospective studies. External validation of our prediction model in independent multicenter cohorts is a priority before clinical implementation. Decision curve analysis should be performed in future validation studies to assess its clinical utility across a range of threshold probabilities; however, due to the current small sample size (n=19), Decision Curve Analysis would be unstable and potentially misleading (39). Beyond clinical and imaging biomarkers, the underlying biology of lymphoma may provide insights into the predisposition to pancreatic infiltration and subsequent pancreatitis. While extensive genomic studies have not revealed specific gene mutations associated with pancreatic localization in lymphoma, the epigenomic landscape of lymphoma represents an intriguing area for future investigation. Epigenetic alterations—including DNA methylation, histone modifications, chromatin remodeling, and noncoding RNAs—play critical roles in B-cell development and lymphomagenesis (40). Whether specific epigenetic signatures predispose certain lymphoma subtypes to extranodal pancreatic involvement remains unknown and warrants further correlative studies. Future investigations incorporating epigenomic profiling of lymphoma tissues from patients with and without pancreatic involvement may identify biomarkers of infiltration risk and potential therapeutic targets (40).

## 5 Conclusions

Lymphoma-associated pancreatitis is rare (1.8% of hematologic malignancies). Treatment-related causes slightly outnumber direct infiltration. EUS-guided tissue acquisition and PET-CT are valuable for differential diagnosis. The nomogram shows preliminary promise in distinguishing treatment-related from non-treatment-related etiology and predicting 1-year mortality. PTCL subtype is the strongest predictor of poor prognosis. However, given the exploratory nature of the model, external validation is required before clinical implementation.

## Data Availability

The raw data supporting the conclusions of this article will be made available by the authors, without undue reservation.
