# Supplementary material for: Lymphoma-associated pancreatitis: a multicenter retrospective cohort study with a prediction model
Source: Front Oncol. 2026 Sep 30;16:1853848. doi: 10.3389/fonc.2026.1853848 (PMC13661464; doi:10.3389/fonc.2026.1853848)
Supplement: Supplementary file 1 [file Table1.docx]

**Supplementary Appendix**

****Supplementary Table S1. Etiological adjudication criteria****

| Etiology category | Diagnostic criteria |
| --- | --- |
| Direct tumor infiltration | Biopsy-proven pancreatic lymphoma/plasmacytoma; OR imaging lesion with typical features (diffuse enlargement, homogenous enhancement) and regression with anti-lymphoma therapy |
| Treatment-related (chemotherapy) | Onset ≤14 days of L-asparaginase, cytarabine, or vinca alkaloids |
| Treatment-related (targeted/immunotherapy) | Onset ≤30 days of BTK inhibitor, ICI, or bispecific antibody initiation |
| Infection-related | Active EBV/CMV viremia (PCR positive) with temporal association |
| Conventional | Gallstones, triglycerides >11.3 mmol/L, corrected calcium >2.75 mmol/L, or heavy alcohol use |
| Multifactorial/idiopathic | Two or more plausible causes, or none identified after thorough workup |

**Supplementary Figure legends**

**Supplementary Figure S1**. ROC curve for the logistic regression model predicting treatment‑related etiology. The dashed diagonal line represents the reference line (AUC = 0.5). The blue solid line represents the ROC curve. AUC = 0.84 (95% CI 0.71–0.96).

**Supplementary Figure S2.** Calibration plot for the etiological classification model. The dashed line represents ideal calibration (predicted = observed). The red solid line represents the bootstrap‑corrected calibration curve. The gray band indicates the 95% confidence interval. Hosmer‑Lemeshow test *p* = 0.38.
